# Unmasking the Silent Liver-Lung Connection: A Pediatric Hepatopulmonary Syndrome Case Report

**DOI:** 10.5811/cpcem.53246

**Published:** 2026-07-10

**Authors:** Shawn A. Haupt, Demetra Lalos, Cornelia Muntean, Dmitriy Vaysman

**Affiliations:** Good Samaritan University Hospital, Department of Pediatrics, West Islip, New York

**Keywords:** hepatopulmonary syndrome, pediatrics, pediatric hypoxia, case report

## Abstract

**Introduction:**

Hepatopulmonary syndrome is a rare but serious cause of pediatric hypoxemia.

**Case Report:**

A 10-year-old obese male with obstructive sleep apnea and asthma presented to the emergency department with low baseline oxygen saturations and exertional hypoxia, despite normal physical exam and outpatient pulmonary function testing. Workup revealed liver cirrhosis, hepatosplenomegaly, gastroesophageal varices, and an elevated alveolar-arterial gradient, raising concern for hepatopulmonary syndrome. He was diagnosed with this disease at a tertiary hepatology center and underwent liver transplantation.

**Conclusion:**

Emergency physicians should consider hepatopulmonary syndrome in children with unexplained hypoxia and liver disease, especially in a child with a normal lung exam and no bronchodilator response.

## INTRODUCTION

Hepatopulmonary syndrome is a severe complication of liver disease and/or portal hypertension resulting in intrapulmonary vascular dilation and hypoxemia. The disease is progressive and often fatal without liver transplantation, which is the only current medical treatment for this disease.^1^ Pediatric hepatopulmonary syndrome is rare; available epidemiological studies suggest that its prevalence in pediatric patients with chronic liver disease is 10–20%.^2^ No specific data is available on the natural history of pediatric hepatopulmonary syndrome without liver transplant; however, a study in adult patients suggests that the median survival time to death is 10.5 months versus 40.8 months in cirrhotic patients without hepatopulmonary syndrome.^3^

The pathophysiology of this rare but serious disease is thought to be due to nitric oxide production leading to intravascular pulmonary dilations, precipitating intrapulmonary arteriovenous shunting and ventilation/perfusion mismatch. Hypoxia occurs as a result.^4^ The onset is often subtle, with patients in the early stages being asymptomatic and developing progressive symptoms of hypoxia and dyspnea as their disease progresses to the late stages. Other symptoms may include worsening dyspnea upon standing (platypnea) and hypoxemia in the upright position (orthodexia). Examination of the patient often reveals clear lungs on auscultation and, depending on severity, may also include digital clubbing, cyanosis, and telangiectasias.^4^,^5^

The diagnosis is established using the following criteria: 1) the presence of liver disease with or without portal hypertension; 2) evidence of intravascular pulmonary dilations established through agitated saline contrast-enhanced transthoracic echocardiography (ECHO); and 3) an arterial-alveolar gradient of > 15 millimeters of mercury (mm Hg).^6^ Because the progressive sequela is devastating, identification as early as possible and intervention is critical in facilitating better survival outcomes.^7^ Existing case reports regarding pediatric hepatopulmonary syndrome are limited due to its rarity.^8^–^11^ To facilitate improved awareness of this disease by emergency physicians and pediatric physicians, we present a case of hepatopulmonary syndrome in a 10-year-old patient who presented with unexplained hypoxemia.

## CASE REPORT

A 10-year-old male patient with a history of morbid obesity, obstructive sleep apnea, and asthma presented by ambulance to the emergency department (ED) from the pulmonologist’s office with a chief complaint of low baseline oxygen saturations at rest and hypoxia with exertion. He was at his pulmonologist’s office for a hospital follow-up after being admitted to the pediatric inpatient floor for an asthma exacerbation three weeks prior when he received nebulized albuterol/ipratropium, nebulized albuterol, and oxygen (O_2_) supplementation via nasal cannula. At the office, he was noted to have peripheral oxygen saturations (SpO_2_) of 92–93% on room air while at rest and an unremarkable physical exam with clear lungs bilaterally. He had normal spirometry with predicted value percentages for forced expiratory volume (FEV_1_), forced vital capacity (FVC), and FEV_1_/FVC ratio at 85%, 83%, and 96%, respectively. His diffusion capacity of the lungs for carbon monoxide was normal, with a predicted value percentage of 96%.

Shortly after he started walking on a treadmill as part of a pulmonary exercise stress test, he desaturated to an SpO_2_ of 83%, prompting the office to call emergency medical services. No medications were given in the office. Emergency medical services arrived and gave him six liters nasal cannula of O_2_ supplementation with improved SpO_2_ to the upper 90s. While en route he seemed more comfortable, and his O_2_ was stopped. On arrival, his vital signs were as follows: heart rate, 102 beats per minute; blood pressure, 127/72 mm Hg; respiratory rate, 28 breaths per minute; oral temperature, 37.1 °C; and SpO_2_, 93% on room air. On further interview, the patient and his mother reported that he often had shortness of breath, both on exertion and when not sick; however, they were unable to elaborate further. The patient admitted to being poorly compliant with his prescribed inhaled fluticasone twice-daily regimen, using it only once a week and not using his prescribed fluticasone nasal spray. He also admitted to having an unhealthy diet.

On examination, he was noted to be morbidly obese with a body mass index of 38 kg/m^2^ (reference range, 15–22 kg/m^2^), well appearing and not in acute distress. His lungs were clear to auscultation with no increased work of breathing, and he had a normal heart auscultation. His abdomen was nontender to palpation, although the assessment for hepatosplenomegaly was limited by body habitus. His oral exam was notable for palatal petechiae and enlarged tonsils, and his skin exam was notable for acanthosis nigricans of the neck and axillary regions.

The patient was given oral dexamethasone, nebulized albuterol, and ipratropium, and we began a diagnostic workup. Given his persistent hypoxemia, despite bronchodilator therapy, normal lung examination, and normal outpatient spirometry, we broadened the evaluation to include systemic (nonpulmonary) causes of hypoxia. Initial laboratory studies, summarized in the table, demonstrated mild leukopenia and moderate thrombocytopenia. Liver chemistries were abnormal with elevated alanine and aspartate transaminases and mildly elevated total and direct bilirubin. Coagulation studies were notable for mildly elevated prothrombin time and partial thromboplastin time. Basic metabolic panel, albumin, D-dimer, troponin, brain natriuretic peptide, and respiratory pathogen panel including COVID-19 polymerase chain reaction were all normal. Arterial blood gas values showed an arterial-alveolar gradient of 36 mm Hg.

*CPC-EM Capsule*

What do we already know about this clinical entity?

*Hepatopulmonary syndrome (HPS) is a rare complication of liver disease that causes intrapulmonary vascular dilation and hypoxemia.*

What makes this presentation of disease reportable?

*This child presented with hypoxemia despite a normal lung exam, normal pulmonary testing, and no response to bronchodilators.*

What is the major learning point?

*In children with unexplained hypoxemia and normal lung findings, consider systemic causes such as liver disease and HPS.*

How might this improve emergency medicine practice?

*Recognizing HPS in the emergency department may reduce anchoring on asthma and expedite hepatology referral and liver transplant evaluation.*

**Image 1:**
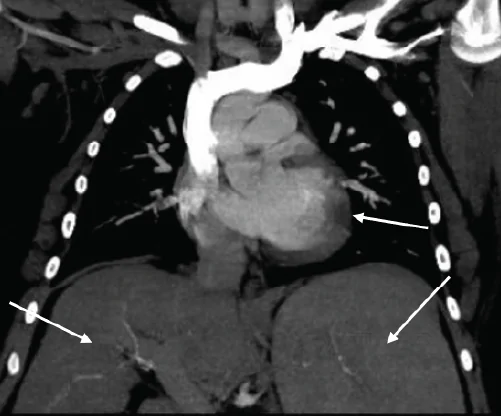
Initial computed tomography angiogram of the chest demonstrating a cardiac silhouette at the upper limits of normal in size (upper arrow) and partial visualization of hepatosplenomegaly (lower left and right arrows).

**Image 2:**
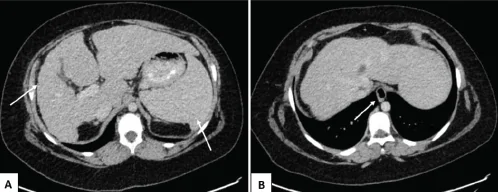
Computed tomography abdomen with intravenous and oral contrast demonstrating: (A) Irregular hepatic contour consistent with cirrhosis and splenomegaly (arrows), suggestive of portal hypertension. (B) Dilated gastroesophageal varices (arrow), consistent with portal hypertension.

Electrocardiogram indicated normal sinus rhythm, and a chest radiograph demonstrated prominent interstitial markings suggestive of reactive airway disease versus mild pulmonary congestion. We performed computed tomography (CT) angiography of the chest to evaluate for pulmonary embolism versus other structural causes of hypoxemia, and incidentally demonstrated gastroesophageal junction varices, a mildly prominent main pulmonary artery concerning for pulmonary artery hypertension, a heart in the upper limits of normal for size, and partial visualization of an enlarged liver and spleen (Image 1), prompting dedicated abdominal imaging. A follow-up CT of the abdomen with oral and intravenous contrast subsequently confirmed hepatosplenomegaly, a nodular capsule consistent with cirrhosis, central intrahepatic biliary duct dilatation, and gastroesophageal varices concerning for portal hypertension (Image 2). Pulmonology was consulted and raised concern for hepatopulmonary syndrome, and the patient was admitted to the inpatient pediatrics floor for further evaluation by cardiology, gastroenterology, and hematology.

While on the pediatrics floor, the patient underwent further investigations. We performed a standard transthoracic echocardiogram (ECHO) and noted no apparent signs of abnormalities, including structural heart disease, abnormal ejection fraction, intracardiac shunts, or pulmonary hypertension. Additional laboratory studies (detailed in the table) to screen for infective etiologies (such as hepatitis A, B and C), autoimmune hepatitis, Wilson disease, alpha-1 antitrypsin deficiency, and celiac disease were negative. The patient was noted to have an elevated immature platelet fraction, which in conjunction with his thrombocytopenia and splenomegaly was suggestive of splenic sequestration of platelets.

During the patient’s three-day inpatient stay, he remained stable on room air with SpO_2_ between 93–95%. Close follow-up was arranged with an outside hospital where pediatric hepatology services were available the day after his discharge for further evaluation, including liver biopsy and other lab investigations. He was subsequently evaluated at the tertiary pediatric hepatology center, where liver biopsy confirmed cirrhosis, and he was diagnosed with hepatopulmonary syndrome. He later underwent liver transplantation. Detailed reports of confirmatory cardiopulmonary shunt testing were not available to us.

## DISCUSSION

The differential for hypoxemia in a pediatric ED patient is broad and includes cardiac, pulmonary, and infective etiologies. Pulmonary causes include the following: obstructive lung diseases such as asthma; restrictive lung diseases such as sarcoidosis, bronchopulmonary dysplasia, or interstitial lung disease; and ventilation/perfusion mismatch diseases such as pulmonary embolism or hepatopulmonary syndrome. Cardiac causes include intracardiac shunts, structural cardiac disease, heart failure, and pulmonary hypertension. Infective causes include viral, bacterial or fungal infections, as well as sepsis, pneumonia, lung empyemas, and bronchiolitis. Hepatopulmonary syndrome would be considered lower on the differential due to its rarity in pediatric patients.

Our patient had a prior diagnosis of asthma and presented with hypoxemia, making asthma expedient as a diagnosis on which to anchor bias. However, he had clear lungs on examination, hypoxia on exertion that did not improve with bronchodilators, and his pulmonary function testing was negative, making obstructive and restrictive causes of lung disease (including asthma) significantly less likely on his differential.^12^ His standard transthoracic ECHO did not show apparent signs of cardiac disease or pulmonary hypertension. His infective and pulmonary embolism workup was negative. He did have an arterial-alveolar gradient of 36 mm Hg on his arterial blood gas value, suggesting an intrapulmonary cause of hypoxia, and with a normal diffusion capacity of the lungs for carbon monoxide. Therefore, it can be inferred that diffusion defects were less likely and a ventilation/perfusion mismatch the most likely mechanism for his hypoxia.^13^ The patient also had significant evidence of liver disease including cirrhosis, elevated liver function tests, and portal hypertension. He was ultimately diagnosed with hepatopulmonary syndrome at a tertiary care center and underwent liver transplantation, supporting the clinical suspicion raised during the initial ED evaluation.

Although liver biopsy confirmed cirrhosis, the exact etiology of the patient’s liver disease remains under evaluation. Multiple etiologies for his cirrhosis, including Wilson disease, infectious and autoimmune hepatitis, alpha-1 antitrypsin deficiency, and celiac disease were ruled out. Given his body habitus, acanthosis nigricans, and poor diet, he showed signs of metabolic syndrome. Non-alcoholic fatty liver disease is the most common etiology of pediatric liver disease and has become more prevalent (approximately 5–10% in children overall and 38–41% in obese children) concurrently with the rise in pediatric obesity globally. A subset of pediatric patients with non-alcoholic fatty liver disease (approximately 20–50%) develop metabolic dysfunction–associated steatohepatitis, in which the presence of steatosis precipitates hepatocellular injury and inflammation, leading to fibrosis, cirrhosis, and liver failure.^14^,^15^ It is certainly possible that this patient may have had metabolic dysfunction–associated steatohepatitis. In children with this disease who develop hepatopulmonary syndrome, liver transplantation is typically required, as occurred in this case.

Although pediatric hepatopulmonary syndrome has been described in the literature, it remains an uncommon and under-recognized cause of hypoxemia in the ED. This case underscores the importance of considering this disease when pulmonary evaluation is unrevealing and the lung examination is normal.

## CONCLUSION

Hepatopulmonary syndrome is a rare but important cause of hypoxemia in children with liver disease and/or portal hypertension. This case highlights the need to broaden the differential diagnosis beyond asthma and other common pulmonary diagnoses when hypoxia persists despite bronchodilators, the lung examination is normal, and pulmonary testing is unrevealing. Early recognition of hepatopulmonary syndrome in the ED can help expedite definitive management, including timely referral for liver transplantation, thus leading to better patient outcomes and decreased mortality rate.

## Figures and Tables

**Table:** Selected laboratory values at presentation to the emergency department and during subsequent inpatient evaluation of a 10-year-old child diagnosed with hepatopulmonary syndrome.

Lab study	Result	Reference range
White Blood Cell Count	4.61 x 10^3^/mcL	5.0–14.0 x 10^3^/mcL
Hemoglobin	15.0 g/dL	11.5–15.5 g/dL
Platelets	53 x 10^3^/mcL	150–400 x 10^3^/mcL
Alanine Transaminase	114 U/L	7–40 U/L
Aspartate Transaminase	118 U/L	13–40 U/L
Total Bilirubin	2.2 mg/dL	0.3–1.2 mg/dL
Direct Bilirubin	0.8 mg/dL	< or = 0.3 mg/dL
Prothrombin	12.3 seconds	9.8–11.8 seconds
Partial Thromboplastin Time	33 seconds	21.4–31.7 seconds
Endomysial Antibody	Negative	Negative
Anti-Liver/Kidney Microscopic Antibody	≤ 20 U	20–25 U (equivocal), > 25 U (positive)
Smooth Muscle Antibody With Reflex Titer	Negative	Negative
Antinuclear Antibody	Negative	Negative
Ceruloplasmin	24 mg/dL	20–50 mg/dL
Alpha-1 Antitrypsin	165 mg/dL	83–199 mg/dL
Gamma-Glutamyl Transferase	57 U/L	< or = 73 U/L
Immature Platelet Fraction	11.5%	0.9–7.0%

*dL*, deciliter; *g*, grams; *L*, liters; *mcL*, microliter; *mg*, milligram; *U*, unit.
